# Clinical Evaluation of a Loop-Mediated Isothermal Amplification (LAMP) Assay for Rapid Detection of *Neisseria meningitidis* in Cerebrospinal Fluid

**DOI:** 10.1371/journal.pone.0122922

**Published:** 2015-04-08

**Authors:** DoKyung Lee, Eun Jin Kim, Paul E. Kilgore, Soon Ae Kim, Hideyuki Takahashi, Makoto Ohnishi, Dang Duc Anh, Bai Qing Dong, Jung Soo Kim, Jun Tomono, Shigehiko Miyamoto, Tsugunori Notomi, Dong Wook Kim, Mitsuko Seki

**Affiliations:** 1 Department of Pharmacy, College of Pharmacy, Hanyang University, Ansan, Republic of Korea; 2 Institute of Pharmacological Research, Hanyang University, Ansan, Republic of Korea; 3 Wayne State University, Eugene Applebaum College of Pharmacy & Health Sciences, Department of Pharmacy Practice, Detroit, Michigan, United States of America; 4 Translational Research Division, International Vaccine Institute, Seoul, Republic of Korea; 5 Department of Bacteriology I, National Institute of Infectious Diseases, Tokyo, Japan; 6 National Institute of Hygiene and Epidemiology, Hanoi, Vietnam; 7 Guangxi Zhuang Autonomous Region Health Bureau, Nanning, Guangxi, China; 8 Chonbuk National University School of Medicine, Jeonju, Korea; 9 Kaneka Corporation, Osaka, Japan; 10 Eiken Chemical Co., Ltd, Tochigi, Japan; 11 Department of Oral Health Sciences, Nihon University School of Dentistry, Tokyo, Japan; 12 Dental Research Center, Nihon University School of Dentistry, Tokyo, Japan; California Department of Public Health, UNITED STATES

## Abstract

**Background:**

*Neisseria meningitidis* (Nm) is a leading causative agent of bacterial meningitis in humans. Traditionally, meningococcal meningitis has been diagnosed by bacterial culture. However, isolation of bacteria from patients’ cerebrospinal fluid (CSF) is time consuming and sometimes yields negative results. Recently, polymerase chain reaction (PCR)-based diagnostic methods of detecting Nm have been considered the gold standard because of their superior sensitivity and specificity compared with culture. In this study, we developed a loop-mediated isothermal amplification (LAMP) method and evaluated its ability to detect Nm in cerebrospinal fluid (CSF).

**Methodology/Principal Findings:**

We developed a meningococcal LAMP assay (Nm LAMP) that targets the *ctrA* gene. The primer specificity was validated using 16 strains of *N*. *meningitidis* (serogroup A, B, C, D, 29-E, W-135, X, Y, and Z) and 19 non-*N*. *meningitidis* species. Within 60 min, the Nm LAMP detected down to ten copies per reaction with sensitivity 1000-fold more than that of conventional PCR. The LAMP assays were evaluated using a set of 1574 randomly selected CSF specimens from children with suspected meningitis collected between 1998 and 2002 in Vietnam, China, and Korea. The LAMP method was shown to be more sensitive than PCR methods for CSF samples (31 CSF samples were positive by LAMP vs. 25 by PCR). The detection rate of the LAMP method was substantially higher than that of the PCR method. In a comparative analysis of the PCR and LAMP assays, the clinical sensitivity, specificity, positive predictive value, and negative predictive value of the LAMP assay were 100%, 99.6%, 80.6%, and 100%, respectively.

**Conclusions/Significance:**

Compared to PCR, LAMP detected Nm with higher analytical and clinical sensitivity. This sensitive and specific LAMP method offers significant advantages for screening patients on a population basis and for diagnosis in clinical settings.

## Introduction

Globally, acute meningitis is a major threat to the health of infants, children, and adults. More than 400 million people live within the “meningitis belt” in sub-Saharan Africa, where epidemic meningitis outbreaks are reported in many countries with almost annual frequency [1]. Bacterial meningitis is responsible for over 1.2 million cases and 135,000 deaths annually [2]. Most bacterial meningitis cases are caused by *Haemophilus influenzae* type b (Hib), *Streptococcus pneumoniae*, or *Neisseria meningitidis* [3]. In addition, these pathogens cause a large number of other invasive diseases, including pneumonia and sepsis. Despite available therapies, meningitis continues to be associated with high rates of mortality and morbidity [4]. Meningitis survivors often suffer from sequelae, including neurological disabilities, hearing loss, and limb loss.

*N*. *meningitidis* is a Gram-negative diplococcal bacterium that infects only humans and causes epidemic meningitis worldwide. On average, meningitis caused by *N*. *meningitidis* results in the death of 1 in every 10 patients [5]. Bacteria first infect the respiratory tract and then invade the central nervous system. The meningococcal polysaccharide capsule is believed to be a virulence factor, as are outer membrane proteins [6]. *N*. *meningitidis* is principally subdivided into 13 serogroups based on the type of polysaccharide in the capsule (A, B, C, D, 29E, H, I, K, L, Y, W-135, X, and Z) [7]. Most cases of invasive disease are caused by strains belonging to serogroups A, B, C, W-135, X, and Y [6, 7].

Time-consuming and resource-intense culturing techniques have been used initially to detect bacteria and to diagnose bacterial meningitis. Bacteria isolated from the CSF of patients are identified by Gram staining and latex agglutination. However, the culture methods take ~36 h [3] and require specific blood-based media and a 5% CO_2_ incubator. Moreover, these methods are influenced by administration of prior antibiotic therapy in patients [8, 9]. To address these problems, gene amplification methods, such as the polymerase chain reaction (PCR), were developed to diagnose bacterial meningitis without requiring culture. However, the equipment required for conventional PCR and real-time PCR assays is relatively expensive, and these techniques are complex to perform in resource-limited laboratory settings in developing countries.

An alternative, rapid, and cost-effective method for gene amplification, the loop-mediated isothermal amplification (LAMP) assay [10] has the potential to overcome the limitations of culture and PCR methods. LAMP employs a DNA polymerase with strand-displacement activity, along with two inner primers (forward inner primer, FIP; backward inner primer, BIP) and outer primers (F3, B3) that recognize six separate regions within a target DNA sequence. This method utilizes a unique priming mechanism that yields specific DNA products in a shorter time than PCR. By using additional primers (i.e., the loop primers: loop primer forward, LF; loop primer backward, LB) designed to anneal the loop structure in LAMP, the LAMP reaction can be accelerated, resulting in enhanced sensitivity [11].

A number of *N*. *meningitidis*-specific genes, such *ctrA*, *crgA*, IS*1106*, 16S rRNA, and *porA*, are commonly used for molecular diagnostic purposes, and PCR-based diagnostic methods targeting those genes have been reported [12]. Two LAMP assays for detecting meningococcus [13, 14] and meningitis bacteria [13, 14] have been developed, but their efficacy in diagnosing bacterial meningitis has not yet been tested using clinical specimens.

In this study, we developed and evaluated a LAMP assay targeting *ctrA* to detect and diagnose *N*. *meningitidis* in CSF specimens from patients with suspected meningitis.

## Methods

### Ethics Statement

We utilized CSF specimens preserved from our previous surveillance study [15]. All CSF specimens utilized in this study were de-identified prior to laboratory processing and analysis. Ethical approvals for patient specimen collection during surveillance were obtained from the following institutions: International Vaccine Institute, Seoul, Korea; Harbor UCLA Medical Center, Torrance, CA, USA; Chonbuk National University Hospital, Jeonju, Korea; Chonju Presbyterian Hospital, Jeonju, Korea; Namwon Medical Center, Namwon, Korea; Jeongeub Asan Foundation Hospital, Jeongeub, Korea; Won Kwang University Hospital, Iksan, Korea; National Institute of Hygiene and Epidemiology, Hanoi, Vietnam; National Institute of Pediatrics, Hanoi, Vietnam; St. Paul Hospital, Hanoi, Vietnam; Bach Mai Hospital, Hanoi, Vietnam; and the Guangxi Zhuang Autonomous Region Center for Disease Control, Nanning, China. Each institution participated in prospective, population-based surveillance for childhood meningitis from 1999 to 2002 [16, 17]. During those surveillance studies, written consent was not obtained as the CSF collection was considered routine standard care for hospitalized children with suspected bacterial meningitis. For this reason, verbal consent of the parent or legal guardian present with the child during the period of hospitalization was recorded in the patient’s medical chart at the time of the clinical lumbar puncture procedure. This consent procedure was approved by the local scientific ethics review committees of the participating institutions.

### Clinical CSF Specimens

Clinicians classified patients as having suspected, probable, or confirmed bacterial meningitis based on clinical signs and symptoms, CSF parameters (e.g., white blood cell count, glucose and protein levels), and bacterial testing (i.e., culture and latex agglutination testing). Children with suspected meningitis who were less than 5 years old were enrolled prospectively by the participating hospitals [15]. CSF was streaked on commercial blood agar culture medium (Becton, Dickinson & Co., Franklin Lakes, NJ), incubated in 5% CO_2_ at 37°C for 3 days, and checked daily for bacterial growth [15]. Isolates were identified using standard microbiological criteria [18]. To compare pneumococcal detection by PCR and LAMP, 1574 CSF specimens (25 PCR-positive and 1549 PCR-negative) were randomly selected from CSF collected between 1998 and 2002 in the prospective study [15] of bacterial meningitis in Korea (*n* = 470), China (*n* = 536), and Vietnam (*n* = 568). CSF specimens were pretreated at 95°C for 3 min and centrifuged at 13000×*g* for 5 min. The supernatant was saved for PCR and LAMP analysis. PCR assays to identify *N*. *meningitidis* were performed as described previously [12]. Aliquots of 2 μL from each CSF specimen were subjected to PCR (reaction volume, 25 μL) and LAMP, as described below.

### Bacterial Strains

This study used 35 standard reference strains comprising 16 *N*. *meningitidis* strains, including serogroups A (HY0001 and NIID1), B (HY0002, H44/76, and NIID2), C (HY0003 and NIID3), D (NIID7), 29-E (NIID8), W-135 (HY0006 and NIID93), X (HY0004 and NIID4), Y (HY0005 and NIID5), and Z (NIID6); 9 non-meningococcal *Neisseria* species, including *N*. *gonorrhoeae* NIID9, *N*. *flavescens* NIID10, *N*. *denitrificans* NIID11, *N*. *elongata* NIID12, *N*. *canis* NIID13, *N*. *cinerea* NIID14, *N*. *lactamica* NIID85, *N*. *mucosa* NIID16, and *N*. *sicca* NIID17; and 10 other bacterial strains, including *S*. *pneumoniae* ATCC 49619, *Staphylococcus aureus* ATCC 29212, *Klebsiella pneumoniae* ATCC 700603, *K*. *oxytoca* ATCC 700324, *Pseudomonas aeruginosa* ATCC 27853, *Escherichia coli* ATCC 25922, *Enterococcus faecalis* ATCC 700324, *Mycobacterium tuberculosis* ATCC 27294, and *H*. *influenzae* ATCC 9007 and IID984.

### Preparation of Chromosomal DNA

Genomic DNA was extracted from those 35 strains by the phenol-chloroform method. For detection limit analysis, genomic DNAs from *N*. *meningitidis* serogroups A (HY0001), B (HY0002), C (HY0003), W-135 (HY0006), X (HY0004), and Y (HY0005) were obtained as described previously [19], and the concentration was determined using a NanoDrop 1000 (Thermo Fisher Scientific Inc., Waltham, MA). Approximately 2.5 fg were taken as one copy of the *N*. *meningitidis* genome, based on the genome of *N*. *meningitidis* serogroup B strain MC58 (accession number AE002098; 2272351 bp [20]). To ascertain the detection limit of the LAMP assay, serial tenfold dilutions of genomic DNA were amplified, and the results were compared with those obtained using conventional PCR.

For the detection limit study, triplicate LAMP testing was performed using tenfold dilutions of genomic DNA. Two technicians tested the same samples independently to confirm the reproducibility of LAMP results. The supernatant of a pooled meningococcus-negative CSF specimen [15] was used for a spiking assay in which serial tenfold dilutions of genomic DNA were amplified, and the results of LAMP and conventional PCR were compared.

### LAMP Primer Design

After alignment analysis, we designed three LAMP primer sets using the LAMP primer support software (Net Laboratory, Kanagawa, Japan) based on published sequences of the *ctrA* (GenBank accession number AE002098), *crgA* (GenBank accession number AF190471), and IS*1106* (GenBank accession number AF422172) genes of *N*. *meningitidis* [12]. Each LAMP primer set included two outer primers (F3 and B3), an FIP, a BIP, and a single loop primer (LF or LB; Table 1 and S1 Table).

**Table 1:** LAMP primers for detection of *N*. *meningitidis*.

The 1st designed	Sequence 5'-3'	bp
ctrA1_F3	AAG AAA TCG GTT TTT CAG CC	20
ctrA1_B3	TAG CGA ATG CGC ATC AGC C	19
ctrA1_FIP^a^	ACA CCA CGC GCA TCA GAA CGT GAA GCC ATT GGC CGT A	37
ctrA1_BIP^b^	TGT TCC GCT ATA CGC CAT TGG TCG TTG GAA TCT CTG CCT C	40
ctrA1_LF	GAT CTT GCA AAC CGC CC	17
The 2nd designed	Sequence 5'-3'	bp
ctrA1_F3	AAG AAA TCG GTT TTT CAG CC	20
ctrA1_B3	TAG CGA ATG CGC ATC AGC C	19
ctrA2_FIP	ACA CCA CGC GCA TCA GAA CTG AAG CCA TTG GCC GTA	36
ctrA1_BIP	TGT TCC GCT ATA CGC CAT TGG TC G TTG GAA TCT CTG CCT C	40
ctrA2_LB	CGT CAG GAT AAA TGG ATT GCT CAA G	25
Nm LAMP	Sequence 5'-3'	bp
ctrA1_F3	AAG AAA TCG GTT TTT CAG CC	20
ctrA1_B3	TAG CGA ATG CGC ATC AGC C	19
ctrA3_FIP	ACA CCA CGC GCA TCA GAA CT**C** ^c^ AAG CCA TTG GCC GTA	36
ctrA1_BIP	TGT TCC GCT ATA CGC CAT TGG TC G TTG GAA TCT CTG CCT C	40
ctrA2_LB	CGT CAG GAT AAA TGG ATT GCT CAA G	25

^a^FIP primer consists of the F1 complementary sequence and F2 sequence.

^b^BIP primer consists of the B1 complementary sequence and B2 sequence.

^c^Mutation (original sequence, G).

### LAMP Reaction

The reaction mixture (25 μL) contained 1.6 μM each of FIP and BIP, 0.2 μM each of F3 and B3, 0.4 μM of LF or LB, 8 U of Bst DNA polymerase large fragment (New England Biolabs, Ipswich, MA), 1.4 mM deoxynucleoside triphosphates, 0.8 M betaine (Sigma, St. Louis, MO), 20 mM Tris-HCl (pH 8.8), 10 mM KCl, 10 mM (NH_4_)_2_SO_4_, 8 mM MgSO_4_, 0.1% Tween 20, and 2 μL of template DNA. The LAMP reactions for *N*. *meningitidis ctrA*, *crgA*, and IS*1106* were performed at 67, 65, and 65°C, respectively, for 60 min and then 80°C for 5 min to terminate the reaction.

### Analysis of LAMP Products

The LAMP reaction was evaluated by visual inspection based on its generation of turbidity proportional to the amount of amplified DNA [21]. For further confirmation, a Loopamp real-time turbidimeter (LA-200 real-time turbidimeter; Eiken Chemical Co., Ltd., Tokyo, Japan) was used to monitor the turbidity in the reaction tube in real-time by reading the OD_650_ every 6 s. We used the application software for the turbidimeter to obtain the amplification time required to exceed a turbidity level of 0.1 (*Tt*), according to the manufacturer’s protocol [21]. For the detection limit study, a colorimetric visual inspection dye (Kaneka, Co., Ltd., Osaka, Japan) dried down in the caps of the reaction tubes was used [22]. After the reactions, the LAMP amplicons were mixed with the dye by inverting the tubes and the color changes were observed.

**Fig 1:**
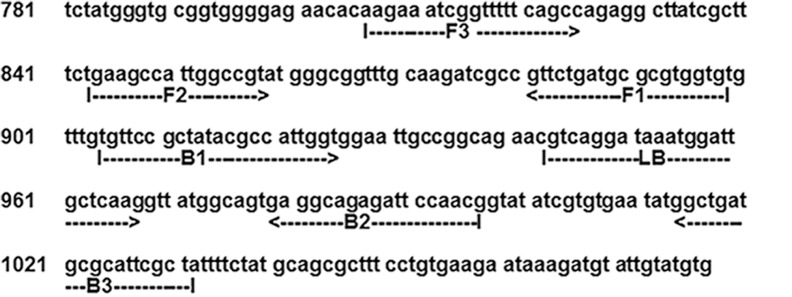
Location of the Nm LAMP primer in the capsular transport gene ctrA (1176 bp).

To verify their structure, the amplified LAMP products were sequenced using a BigDye Terminator v. 3.1 cycle sequencing kit (Applied Biosystems, Foster City, CA) and an ABI PRISM 377 DNA sequencer (Applied Biosystems) according to the manufacturer’s instructions. The target region was between F2 and B2, and the primer sequences were from the F2 and B2 regions (Fig 1).

### Checking Nonspecific LAMP Amplification

To facilitate detection of nonspecific amplification even under stringent conditions, the primer set was left at room temperature for 30 min before the reaction, and then we assessed the occurrence of nonspecific amplification after a reaction time of 90 min. We used seven negative controls (distilled water). The reactions were observed by means of the Loopamp real-time turbidimeter (Eiken Chemical Co., Ltd., Tokyo, Japan).

### PCR Assay

To identify *N*. *meningitidis*, conventional PCR primers described previously for *ctrA* (Nm PCR) were used [12]. The primer sequences were *ctrA*_F, GCT GCG GTA GGT GGT TCA A; *ctrA*_R, TTG TCG CGG ATT TGC AAC TA. The PCR mixture (25 μL) consisted of 0.2 mM of each deoxyribonucleoside triphosphate, 10 mM Tris-HCl buffer (pH 8.0), 100 mM KCl, 25 mM MgCl_2_, 2.5 U of Ex Taq DNA polymerase (TaKaRa Bio Inc., Otsu, Japan), 0.4 μM of each primer, and 2 μL of template DNA.

PCR was performed as follows: initial denaturation at 94°C for 5 min, followed by 30 cycles of denaturation at 94°C for 1 min, annealing at 58°C for 30 s, and extension at 72°C for 1 min, with a final extension at 72°C for 10 min in a T100 Thermal Cycler (Bio-Rad Inc., Hercules, CA). Products were visualized by resolution on a 2% agarose gel followed by staining with ethidium bromide.

### Statistical Analysis

The clinical sensitivity, specificity, positive predictive value (PPV), and negative predictive value (NPV) of conventional PCR testing were compared to those of LAMP (PCR was taken as the gold standard).

## Results & Discussion

**Fig 2:**
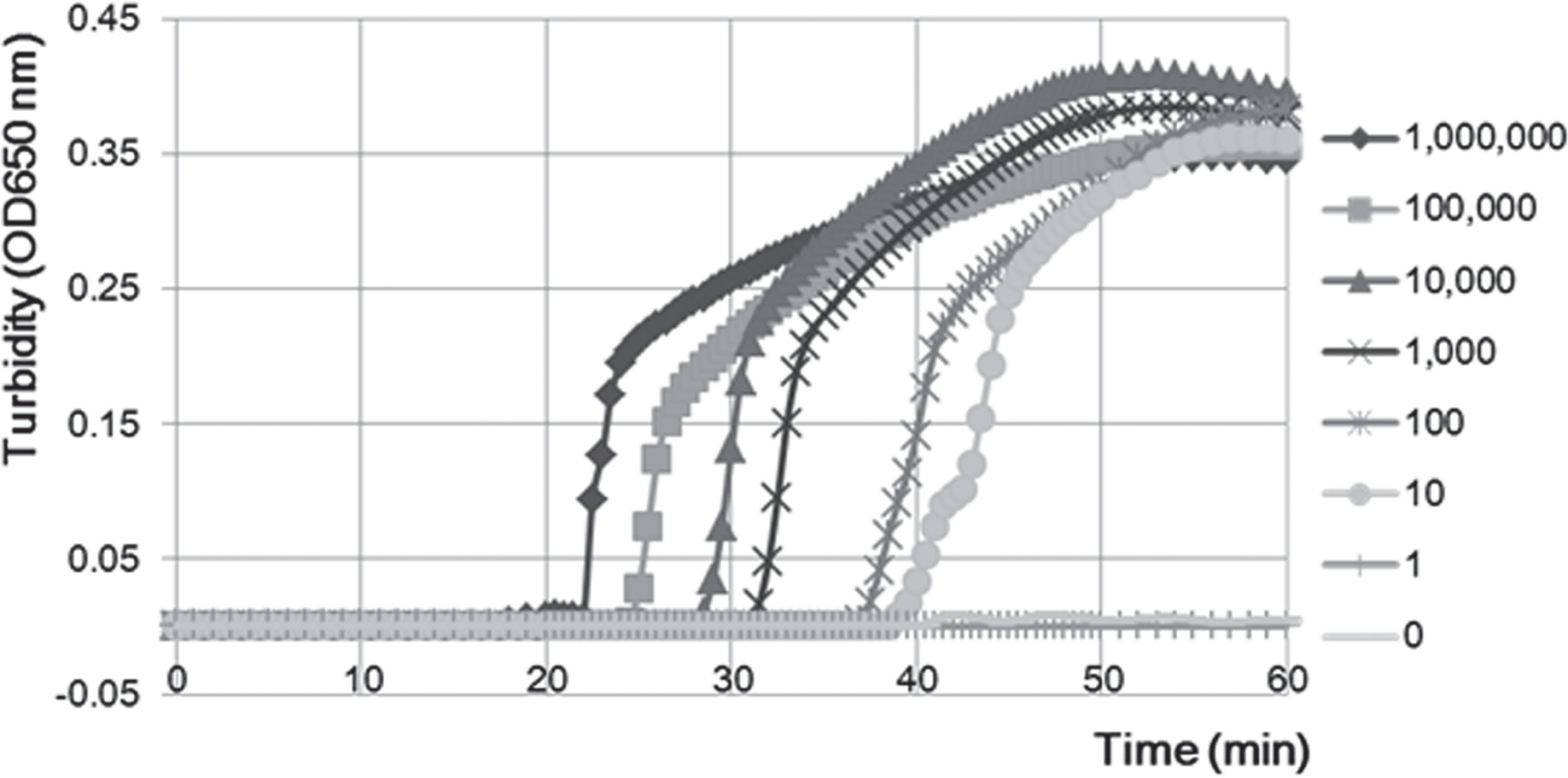
Real-time monitoring of Nm LAMP reaction mixture turbidity. *N*. *meningitidis* serogroup B reference DNA; dilution series from 1,000,000 copies to 1 copy. The molecular weight of the genomic DNA is 2.2 Mbp. The molecular weight of one copy is 2.5 fg. The LAMP assay detected 10 copies of *N*. *meningitidis* serogroup B within 60 min.

**Fig 3:**
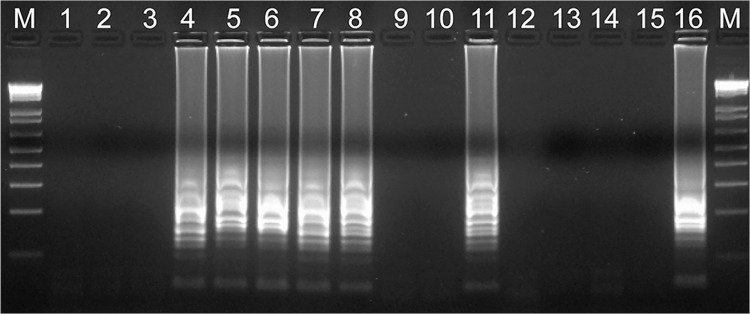
Electrophoretic analysis of Nm LAMP products. The Nm LAMP products showed a ladder-like pattern on 2% gel electrophoresis and ethidium bromide staining. Lane M, molecular size marker (100-bp ladder; New England Biolabs, Beverly, MA); lanes 1–3, 9, 10, and 12–14: clinical samples (negative for *N*. *meningitidis*); lanes 4–8 and 11: clinical samples (positive for *N*. *meningitidis*); lane 15: negative control; lane 16: positive control

We developed a rapid and sensitive LAMP reaction for detecting *N*. *meningitidis* that reduced the occurrence of nonspecific reactions. Using the primer set we developed, the LAMP assay successfully amplified the 194-bp target sequence of the *ctrA* gene within 45 min (Fig 2). No nonspecific amplification reactions were observed within 90 min even under stringent conditions (Table 2). The amplified product was visible on an agarose gel (Fig 3) and showed a ladder-like pattern that was characteristic of the LAMP reaction, indicating the production of stem-loop DNAs with inverted repeats of the target sequence [10].

**Table 2:** Nonspecific LAMP reaction test using *ctrA* primer sets.

	First designed^a^	Second designed^b^	Nm LAMP^c^
Reaction temperature	65°C	67°C	67°C
Trial no. 1^d^	58.1 min^e^	^f^	
2	87.4		
3		49.2	
4	78.4		
5			
6			
7			

^a^Amplification reaction using the first designed *ctrA* primer set.

^b^Amplification reaction using the second designed *ctrA* primer set.

^c^Amplification reaction using the final *ctrA* primer set with a mutation in FIP (Nm LAMP).

^d^A sample of each trial used distilled water.

^e^Detection time determined by a Loopamp real-time turbidimeter.

^f^ —, no false positive reaction.

.

### Reducing Nonspecific LAMP Amplification

Most nucleic acid amplification assays, including PCR, yield nonspecific results because of the extended reaction time or the number of reaction cycles. In clinical settings, such nonspecific reactions can result in misdiagnoses.

Table 2 shows the nonspecific amplification detection times of three primer sets. Some nonspecific amplification reactions occurred within 90 min when the first and second (FIP and loop-primer modified) *ctrA* primer sets were used. After adding a single mutation to the modified FIP primer sequence, no nonspecific amplification occurred within a 90-min reaction time (we named the final *ctrA* primer set Nm LAMP, Table 1). To confirm our findings, we also tested nonspecific reactions using a 180-min reaction time (the normal reaction time is 60 min). Within 180 min, some nonspecific amplification reactions occurred using the first and second *ctrA* primer sets, but none occurred using the final *ctrA* LAMP primer set (Nm LAMP, S2 Table). Moreover, the detection limit of Nm LAMP was lower than that using the previous *ctrA* LAMP primer sets (10 vs. 100 copies).

Some primer sets used for LAMP assays can cause nonspecific amplification because of interactions between or within primers. The LAMP primer-design software Primer Explorer (http://primerexplorer.jp/) has a dimer check function, which allows interactions between primers to be avoided. However, interactions within primers are not considered in this software. Indeed, LAMP uses two inner primers of 40–50 bp (FIP and BIP); such long primer sequences tend to result in nonspecific reactions.

To detect nonspecific amplification even under stringent conditions, the primer set was left at room temperature (25°C) for 30 min. Then we assessed the occurrence of nonspecific amplification reactions during a 90-min reaction time. After notification of nonspecific reaction, we removed each primer (F3, B3, FIP, BIP, LF, LB) sequentially from the LAMP primer set and performed LAMP reactions under the same conditions. Nonspecific reactions occurred when all primers were included but not after removal of the FIP or BIP primer from the LAMP primer set.

To identify hairpin structures, we calculated the amount of free energy (ΔG), which influences the frequencies of primer dimer and nonspecific reactions, by using the mfold Web Server (http://mfold.rna.albany.edu/?q=mfold/RNA-Folding-Form). A lower ΔG increases the frequencies of primer dimer formation and nonspecific reactions. However, addition of a single mutation (C to G) in the middle of the FIP primer sequences (Table 1) increased the ΔG (minimum ΔG = –6.00 to—2.20 kcal/mol in FIP), enabling nonspecific reactions to be avoided.

### Analytical Specificity of Nm LAMP

To evaluate the species specificity of the Nm LAMP primer set, we tested 35 reference strains, as described above. For each assay mixture, 10^6^ copies of genomic DNA of each strain were used. In the Nm LAMP reaction, meningococcal DNA was amplified within 30 min. In contrast, genomic DNA of the non-meningococcal strains was not amplified even after 60 min of incubation.

Amplified products were sequenced, and the sequences were compared with those of the targeted region (bases 860–978) of the meningococcus *ctrA* gene (between F2 and B2, Fig 1). The sequences obtained were identical to those expected (S1 Fig).

### Detection Limit of the Nm LAMP Assay

Meningococcal genomic DNA was reliably amplified to a lower limit of 10 genome copies per reaction (25 microliter of reaction mix, triplicate trials) (Table 3). Moreover, we confirmed amplification of as little as one genome copy by the Nm LAMP assay. In contrast, the PCR assay targeting the *ctrA* gene had a detection limit of 10^4^ genome copies per reaction (Table 3). By using CSF specimens spiked with *N*. *meningitidis* serogroup B DNA, we found that the detection limit of the Nm LAMP assay was identical at 10 genome copies per reaction, compared with 10^5^ genome copies per reaction for PCR (Table 3). Therefore, the detection limit of the Nm LAMP assay was 10^3^–10^4^-fold lower than that of PCR.

**Table 3:** Detection limits of Nm LAMP and PCR assays targeting the *ctrA* gene and using Nm-serogroup-B-spiked CSF specimens.

*N*. *meningitis* serogroup B genome copy number using the *ctrA* gene ^a^ ^,^ ^b^								
Assay	10^6^	10^5^	10^4^	10^3^	10^2^	10	1	0
PCR	+	+	+	–	–	–	–	–
LAMP	+	+	+	+	+	+	±	–
*N*. *meningitis* genome copy number using Nm-serogroup-B-spiked CSF specimens^a^ ^,^ ^b^ ^,^ ^c^								
PCR	+	+	–	–	–	–	–	–
LAMP	+	+	+	+	+	+	±	–

^a^ PCR results were obtained by electrophoresis. LAMP results were obtained by visual inspection of turbidity, colorimetric visual inspection dye, and by using a real-time turbidimeter.

^b^ +, Amplification;–, No amplification; ±, amplification occurred once.

^c^ Duplicate results.

**Fig 4:**
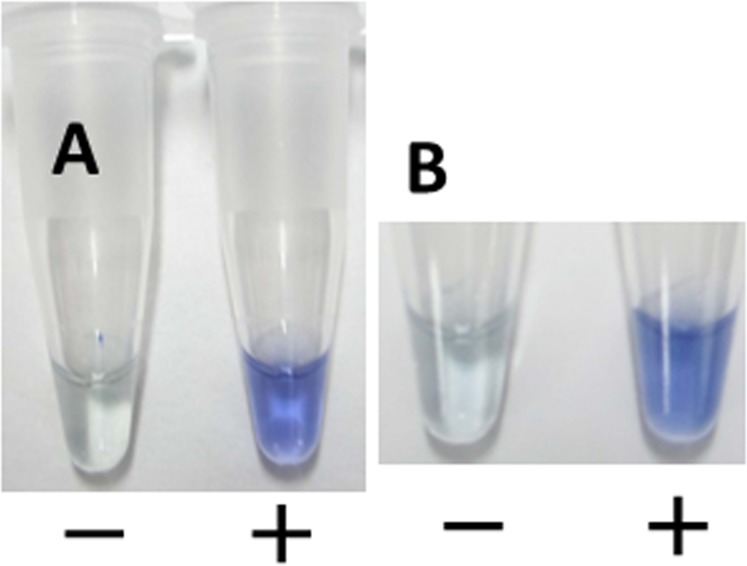
Visual-inspection of dye-mediated monitoring of the Nm LAMP assay. (A) The original pale-yellow color of the visual inspection dye (Kaneka, Co., Ltd, Osaka, Japan) changed to blue in the case of a positive reaction; in the case of a negative reaction, the original pale-yellow color was retained. (B) Visual observation after 7 days.

The detection limit of the Nm LAMP assay was confirmed by using a Loopamp real-time turbidimeter, by direct visual inspection, or by adding colorimetric visual inspection dye (Kaneka, Co., Ltd.) to the test tube. Although there was no difference in detection limits of the three assays, better and easier-to-understand visualization was obtained by using a colorimetric dye. This was the first clinical evaluation of colorimetric visual inspection dye. Using CSF specimens spiked with *N*. *meningitidis* serogroup B, we observed that the initial pale-yellow color of the dye changed to blue, which could be observed under natural light without the need for UV light. If no amplification occurred, the dye retained its pale-yellow color. This change in color remained for at least 1 week (Fig 4).

Goto *et al*. reported a different colorimetric LAMP method that used hydroxy naphthol blue [23]. However, using that method, differentiating positive and negative reactions was problematic because of the similarity of the colors generated (violet and blue). The new colorimetric visual inspection dye reported here easily differentiates positive and negative samples, which will facilitate its use clinically, especially in resource-limited settings in developing countries.

### Nm LAMP and PCR Analysis of CSF Specimens

We tested 1574 CSF specimens using the Nm LAMP and PCR assays. Using the Nm LAMP assay, *N*. *meningitidis* was detected in 20 specimens from Vietnam, 5 from China, and 6 from Korea. Among the 31 Nm LAMP-positive specimens, 25 (80.6%) were positive and 6 were negative by Nm PCR, and 3 CSF specimens were *N*. *meningitidis* culture positive (9.7%) (Table 4). Thus, the culture method had low sensitivity and is inappropriate for use in diagnosis of bacterial meningitis. In addition, 1543 Nm LAMP-negative specimens (100%) were also negative by the Nm PCR and culture methods (Table 5). All three *N*. *meningitidis* culture-positive specimens (100%) were positive by both the Nm LAMP and PCR assays.

**Table 4:** Comparison of diagnostic methods for clinical specimens.

Country	Meningococcus detection assay		
	Culture	PCR	LAMP
Vietnam (n = 568)	3^a^	15	20
China (n = 536)	0	5	5
Korea (n = 470)	0	5	6
Total (n = 1574)	3	25	31

^a^Number of positive results.

**Table 5:** A comparative analysis of PCR and Nm LAMP detection of *N*. *meningitidis*.

Nm LAMP	PCR positive	PCR negative	Total	Sensitivity (%)	Specificity (%)	PPV (%)	NPV (%)
positive	25	6	31	100.0	99.6	80.6	100.0
negative	0	1543	1543				
Total	25	1549	1574				

In order to confirm the Nm PCR results, we performed an additional PCR experiment using the outer primers (F3/ B3) for Nm-LAMP (Table 1). The results were identical to those of the Nm PCR primer set. Thus, we do not think a mutation of the Nm PCR primer binding region influenced the results using the 6 Nm LAMP-positive/Nm PCR-negative clinical CSF specimens.

Using LAMP assays that target *crgA* and *IS1106* (S1 Table), we confirmed that the Nm LAMP-positive CSF samples were also positive for these two genes (data not shown). By sequencing, we confirmed that all of the Nm LAMP products from CSF corresponded to the selected gene target.

Compared with PCR, the Nm LAMP test had a clinical sensitivity of 100%, clinical specificity of 99.6%, PPV of 80.6%, and NPV of 100%. Thus, the Nm LAMP assay was more sensitive than the PCR method and has very high negative predictive potential.

In our analysis, 2% (28/1571) of the culture-negative CSF specimens and 0.4% (6/1549) of the PCR-negative CSF specimens were Nm LAMP-positive. From a clinical viewpoint, the excellent clinical sensitivity, clinical specificity, PPV, and NPV of the Nm LAMP assay will facilitate diagnosis of meningococcal infection. More accurate diagnosis of *N*. *meningitidis* infection using this Nm LAMP assay will improve the use of antibiotics and reduce the currently high levels of antibiotic resistance. From a public health standpoint, widespread application of the Nm LAMP assay could yield higher detection rates and alter global meningococcal prevalence rates [19].

The higher clinical sensitivity of the Nm LAMP assay found here is consistent with reports of LAMP assays that detect *H*. *influenzae* type b or *S*. *pneumoniae* in CSF [24, 25]. Previous studies demonstrated that the LAMP reaction is more tolerant of potentially perturbing biological substances than PCR [26]. The main reason for the higher clinical sensitivity of the LAMP assay is its higher analytical sensitivity.

In addition, the robust performance of the LAMP assay in previous studies [27] suggests that LAMP-based detection of *N*. *meningitidis* and other invasive bacterial pathogens would be feasible in a wide variety of clinical settings. In our laboratory, the per-specimen cost (including costs of reagents, supplies, and personnel time) of the Nm LAMP assay is markedly lower than that of PCR. The low technology requirements for the LAMP assay suggest that this platform would be particularly suitable for resource-limited settings in developing countries.

In conclusion, the Nm LAMP assay was more sensitive than the previously described PCR method [12]. The lower detection limit of the Nm LAMP assay yielded a higher detection rate in CSF specimens than the PCR assay. Further evaluation of Nm LAMP in prospective studies is now underway to confirm its clinical sensitivity, clinical specificity, predictive values, and likelihood ratios compared with bacterial culture, antigen detection, and PCR.

## Supporting Information

S1 Fig Sequence data of Nm LAMP amplified products. (TIF)

S1 Table LAMP primers that target *crgA* and *IS1106* for detecting *N*. *meningitidis*. (DOC)

S2 Table Nonspecific LAMP reaction test using the designed *ctrA* primer sets (reaction time, 180 min). (DOC)
