# Correction: Clinical Evaluation of a Loop-Mediated Isothermal Amplification (LAMP) Assay for Rapid Detection of *Neisseria meningitidis* in Cerebrospinal Fluid

**DOI:** 10.1371/journal.pone.0130506

**Published:** 2015-06-17

**Authors:** The PLOS ONE Staff

Table 2 features blank entries where dashes should be featured instead. Please view the correct Table 2 here.

**Table 2:** Nonspecific LAMP reaction test using *ctrA* primer sets.

	First designed^a^	Second designed^b^	Nm LAMP^c^
Reaction temperature	65°C	67°C	67°C
Trial no. 1^d^	58.1 min^e^	-^f^	-
2	87.4	-	-
3	-	49.2	-
4	78.4	-	-
5	-	-	-
6	-	-	-
7	-	-	-

^a^Amplification reaction using the first designed *ctrA* primer set.

^b^Amplification reaction using the second designed *ctrA* primer set.

^c^Amplification reaction using the final *ctrA* primer set with a mutation in FIP (Nm LAMP).

^d^A sample of each trial used distilled water.

^e^Detection time determined by a Loopamp real-time turbidimeter.

^f^-, no false positive reaction.
