# Supplementary figures and images for: Clinical Evaluation of a Loop-Mediated Isothermal Amplification (LAMP) Assay for Rapid Detection of Neisseria meningitidis in Cerebrospinal Fluid
Source: PLoS One. 2015 Apr 8;10(4):e0122922. doi: 10.1371/journal.pone.0122922 (PMC4390149; doi:10.1371/journal.pone.0122922)

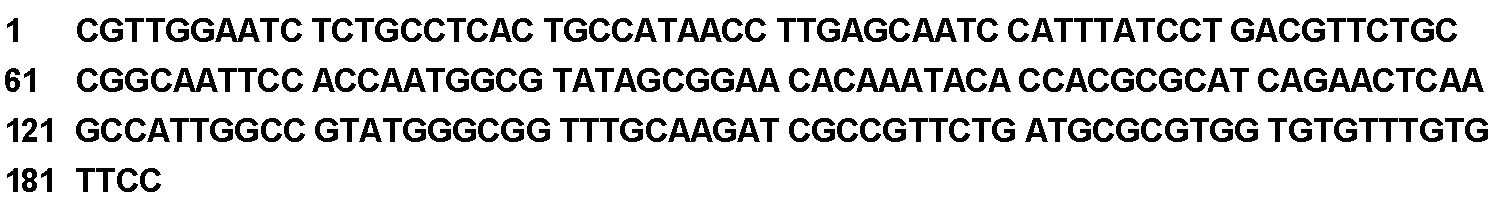

Supplement: S1 Fig — (TIF) [file pone.0122922.s001.tif]
