# Supplementary material for: Clinical Evaluation of a Loop-Mediated Isothermal Amplification (LAMP) Assay for Rapid Detection of Neisseria meningitidis in Cerebrospinal Fluid
Source: PLoS One. 2015 Apr 8;10(4):e0122922. doi: 10.1371/journal.pone.0122922 (PMC4390149; doi:10.1371/journal.pone.0122922)
Supplement: S1 Table — (DOC) [file pone.0122922.s002.doc]

**Table S1.** LAMP primers that target *crgA* and *IS1106* for detecting *N. meningitidis*

| *crgA* LAMP | Sequence 5'-3' | bp |
| --- | --- | --- |
| *crgA*_F3 | CCG GAG AAT TGG ACG ATT CC | 20 |
| *crgA* _B3 | GCG GTA AAG TGC GGT GAA | 18 |
| *crgA* _FIP^a^ | CGT GCC GTG TTT TGC CAG GTA TAC GCC ATC TGT TTG ACA GC | 44 |
| *crgA* _BIP**^b^** | CTA CAG AAG AGC TTG CCG GTC ACG CAT CTA AAA CCG CCC AT | 44 |
| *crgA* _LF | ACT GGC GAT TAC GCG GAA G | 19 |
| *IS1106* LAMP | Sequence 5'-3' | bp |
| *IS1106*_F3 | CCG TTT CCC GCT ATT GAA GT | 20 |
| *IS1106*_B3 | AAT CGA TGC GGG TGA TGA G | 19 |
| *IS1106*_FIP | CGC GGT GGT CTC TAA GGT AAC GTG GAC CAG GTG ATT GAT TGG | 42 |
| *IS1106*_BIP | CTG CTG TCC ATG TTC AAA GCC GTT CGA GTT CGG GAT CGG AG | 41 |
| *IS1106*_LB | TCC TGC TCG GAC AAT GGC AC | 20 |

^a^FIP primer consists of the F1 complementary sequence and F2 sequence.

^b^BIP primer consists of the B1 complementary sequence and B2 sequence.
