# Supplementary material for: Clinical Evaluation of a Loop-Mediated Isothermal Amplification (LAMP) Assay for Rapid Detection of Neisseria meningitidis in Cerebrospinal Fluid
Source: PLoS One. 2015 Apr 8;10(4):e0122922. doi: 10.1371/journal.pone.0122922 (PMC4390149; doi:10.1371/journal.pone.0122922)
Supplement: S2 Table — (DOC) [file pone.0122922.s003.doc]

**Table S2.** Nonspecific LAMP reaction test using the designed *ctrA* primer sets (reaction time, 180 min).

|  | First designed^a^ | Second designed^b^ | Nm LAMP^c^ |
| --- | --- | --- | --- |
| Reaction temperature | 65℃ | 67℃ | 67℃ |
| Detection time (min) |  |  |  |
| Trial no. 1 ^d^ | 109.6^e^ | 139.4 | ―^g^ |
| 2 | 125.2 | 170.9 | ― |
| 3 | 117.3 | ― | ― |
| Positive control^f^ | 16.7 | 22.8 | 21.1 |

^a^Amplification reaction using the first designed *ctrA* primer set.

^b^Amplification reaction using the second designed *ctrA* primer set.

^c^Amplification reaction using the final *ctrA* primer set with a mutation in FIP (Nm LAMP).

^d^ A sample of each trial consisted of distilled water.

^e^ Detection time determined by a Loopamp real-time turbidimeter.

^f^ Positive control, sample was genomic DNA of Nm serogroup B (10^6^ copies per reaction tube).

^g^ ―, no false positive reaction.
